# Effects and Mechanisms of Resveratrol on Aging and Age-Related Diseases

**DOI:** 10.1155/2021/9932218

**Published:** 2021-07-11

**Authors:** Dan-Dan Zhou, Min Luo, Si-Yu Huang, Adila Saimaiti, Ao Shang, Ren-You Gan, Hua-Bin Li

**Affiliations:** ^1^Guangdong Provincial Key Laboratory of Food, Nutrition and Health, Department of Nutrition, School of Public Health, Sun Yat-sen University, Guangzhou 510080, China; ^2^Research Center for Plants and Human Health, Institute of Urban Agriculture, Chinese Academy of Agricultural Sciences, Chengdu 610213, China; ^3^Key Laboratory of Coarse Cereal Processing (Ministry of Agriculture and Rural Affairs), Sichuan Engineering & Technology Research Center of Coarse Cereal Industrialization, Chengdu University, Chengdu 610106, China

## Abstract

The aging of population has become an issue of great concern because of its rapid increase. Aging is an important risk factor of many chronic diseases. Resveratrol could be found in many foods, such as grapes, red wine, peanuts, and blueberries. Many studies reported that resveratrol possessed various bioactivities, such as antioxidant, anti-inflammatory, cardiovascular protection, anticancer, antidiabetes mellitus, antiobesity, neuroprotection, and antiaging effects. The antiaging mechanisms of resveratrol were mainly ameliorating oxidative stress, relieving inflammatory reaction, improving mitochondrial function, and regulating apoptosis. Resveratrol could be an effective and safe compound for the prevention and treatment of aging and age-related diseases. In this review, we summarize the effects of resveratrol on aging, life extension, and several age-related diseases, with special attention paid to the mechanisms of antiaging action.

## 1. Introduction

The expansion in the aged population has emerged as a matter of great concern. Over the past 60 years, the proportion of the world population aged 60 and over had experienced only a slight increase, from 8% to 10%. However, in the next 40 years, this group is estimated to soar to 22% of the total population, jumping from 0.8 billion to 2 billion [1]. In the progress of aging, living organisms experienced a series of progressively degenerative changes and became more sensitive to the internal and external stimuli, leading to aggravation of oxidative stress, accumulation of inflammation, apoptosis of cells, and damage to structures and functions of cells and organs [2–4]. Hence, as age is growing, the risks of susceptibility to various diseases (such as neurodegenerative diseases, sarcopenia, cardiovascular diseases, diabetes mellitus, obesity, and malignant tumors) and vulnerability to death are increased [5–7]. In this respect, a challenge facing the aging population is to decrease the impact of aging on health and avoid the occurrence and development of age-related diseases. The plethora of literature has supported that bioactive compounds from traditional Chinese medicine or natural products could be an effective and safe strategy to protect against aging and age-related diseases [8–12].

Polyphenols are bioactive compounds that extensively existed in plant foods and have many health-promoting effects by the different mechanisms, such as antioxidation, anti-inflammation, immunomodulation, and modulating gut microbiota [11, 13]. Resveratrol is a natural phenolic compound and has been found in many foods, such as grapes, peanuts, blueberries, and red wine. Resveratrol has shown many bioactivities, such as antioxidant, anti-inflammatory, immunomodulatory, hypotensive, and hypolipidemic actions, as well as efficacies in the prevention and management of cancer, cardiovascular diseases, neurodegenerative diseases, and obesity [14, 15]. In addition, many studies highlighted its importance in aging treatment through suppressing oxidative stress, inhibiting inflammatory response, improving mitochondrial function, and modulating apoptosis [16, 17]. Moreover, the interaction between resveratrol and gut microbiota in the protection of health has attracted increasing attention in recent years [18]. In this review, we summarize the effects of resveratrol on antiaging, lifespan, and several age-related diseases (neurodegenerative diseases, sarcopenia, cardiovascular diseases, and malignant tumors), and the mechanisms of antiaging are paid special attention.

## 2. Effect of Resveratrol on Life Extension

Longevity is a vital target of antiaging research and an important index reflecting the antiaging efficacy [19]. Accumulating studies revealed that resveratrol could extend lifespan (Table 1). For example, a meta-analysis indicated that resveratrol acted as a life-extending agent, concluding from 19 published papers with six species (yeast, nematodes, mice, fruit flies, Mexican fruit flies, and turquoise killifish) [20]. Autophagy could promote the clearance of impaired cellular organelles (mitochondria, endoplasmic reticulum, etc.) and dysfunctional proteins, which contributes to the life extension and antiaging effect [21, 22]. A study demonstrated that resveratrol could induce autophagy in human cells *in vitro* and *Caenorhabditis elegans in vivo* and prolong lifespan in *Caenorhabditis elegans*, while this effect was prevented under the condition of knockdown or knockout of sirtuin (Sirt)1. The results suggested that resveratrol promoted longevity through the Sirt1-dependent induction of autophagy [23]. Another study compared the effect of thymol and resveratrol on the life span of honey bees and found that the bees fed with resveratrol syrup lived longer (25 days) than those fed with thymol or control syrup (23 and 20 days, respectively) [24]. Moreover, a study evaluated the difference in adult longevity of short-lived *Drosophila melanogaster* populations fed with different concentrations of resveratrol. Resveratrol supplementation prolonged adult longevity in both the male and female flies in a nonlinearly dose-dependent manner by scavenging reactive oxygen species (ROS) and neuroprotection, without affecting reproduction [25]. In a study, a transgenic resveratrol rice DJ526, which had 180 times more resveratrol than the conventional grain, remarkably prolonged the median longevity of *Drosophila melanogaster* by up to 50% in contrast to the control. It also improved age-related symptoms, including locomotive deterioration, body weight gain, eye degeneration, and neurodegeneration [26]. Another study showed that the supplement of resveratrol extended the lifespan of *Nothobranchius guentheri* (a wild type of annual fish) but had no effect on their body size. Resveratrol-treated fish exhibited better cognitive and locomotor activities than the fish in the control group, and resveratrol retarded the aging-related histological markers, including the expression of senescence-associated *β*-galactosidase activity and lipofuscin formation [27]. In addition, a study used HtrA2 knockout mice to evaluate the effect of resveratrol on lifespan, which usually developed neuromuscular abnormalities around postnatal day 23 and died around day 28. The results showed that oral administration of resveratrol increased the median survival of the mice by 10 days (from 32 days to 42 days) [28]. However, inconsistent evidence existed. For example, a study pointed out that red wine and equivalent oral pharmacological doses of resveratrol could delay vascular aging but had no effect on lifespan extension in rats [29].

To sum up, resveratrol could extend lifespan in many animal models mainly by inducing autophagy, reducing oxidative stress and neuroprotection. In addition, some fruits and vegetables, such as grape, peanut, blueberry, cucumber, tomato, red cabbage and spinach, have relatively high contents of resveratrol, and their effects on lifespan should be evaluated in the future.

## 3. Effect of Resveratrol on Age-Related Diseases

**Figure 1:**
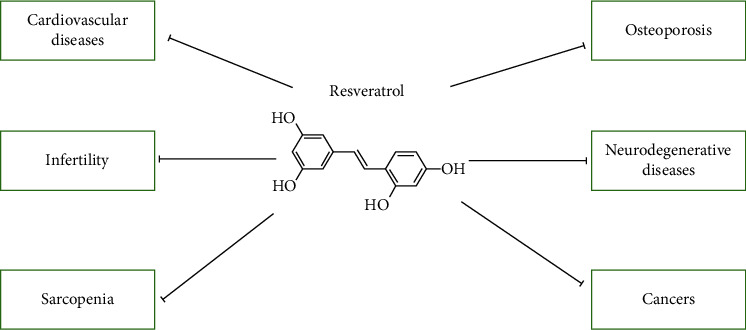
The effects of resveratrol on age-related diseases. Resveratrol could protect against age-related diseases, such as neurodegenerative diseases, cardiovascular diseases, sarcopenia, infertility, osteoporosis, and cancers.

Lowering the risk of age-related diseases is another crucial goal of antiaging research. Here, we summarized the effects of resveratrol on several age-related diseases (Table 2 and Figure 1).

### 3.1. Effect of Resveratrol on Neurodegenerative Diseases

Aging is reported to be the primary risk factor of neurodegenerative diseases. Aging is accompanied by neuroinflammation, autophagy dysregulation, neuronal apoptosis, and elevated oxidative status, which leads to progressive memory loss and motor impairment. It eventually increases the risk of neurodegenerative diseases, such as Alzheimer's disease, Parkinson's disease, and dementia [30, 31]. The plethora of literature showed the protection of resveratrol against neurodegenerative diseases. For example, a study revealed the protection of resveratrol treatment on hippocampal plasticity and memory performance in female Balb/C mice. Resveratrol induced neuronal differentiation in adult hippocampal precursor cells without affecting the proliferation *in vitro*. Also, resveratrol intervention improved behavioral performance, increased the production of new neurons, elevated the population of doublecortin-expressing intermediate cells, and promoted hippocampal neurogenesis *in vivo*. The resveratrol-treated mice showed an increase in the levels of phosphoprotein kinase B (Akt) and phosphoprotein kinase C, indicating the involvement of these signaling pathways [32]. Furthermore, after intraventricular injection of resveratrol for 7 days, the long-term memory formation and the long-time potentiation induction from hippocampus CA1 were improved in C57BL/6J mice, while these effects were blocked in Sirt1 mutant mice [33]. In another study, aged male F344 rats were treated with resveratrol or vehicle for 4 weeks. The results found that resveratrol-treated animals showed the improved learning, memory, and mood functions. Resveratrol also increased net neurogenesis and microvasculature, decreased astrocyte hypertrophy, and microglial activation in the hippocampus [34]. Moreover, a study evaluated the effects of chronic administration of resveratrol on aged male Wistar rats by testing tryptophan hydroxylase (TPH) and tyrosine hydroxylase (TH) activities (mediating central monoaminergic neurotransmitter synthesis) and hippocampal-dependent working memory. The results reported that resveratrol reversed an age-dependent decline in cognitive functions through enhancing the secretion of neurotransmitters, including serotonin, noradrenaline, and dopamine. These changes were largely due to the increased activities of TPH and TH [35]. Additionally, another study compared the difference between the young and old rats treated with or without resveratrol. The results found that old rats showed emotional and spatial learning memory damage compared to young rats, while resveratrol could reverse the cognitive impairment via inhibiting the production of inflammatory cytokines [36].

In short, resveratrol showed protective effects against neurodegenerative diseases by enhancing the secretion of neurotransmitters, increasing the production of new neurons, decreasing neuroinflammation and oxidative stress, and promoting hippocampal neurogenesis.

### 3.2. Effect of Resveratrol on Cardiovascular Diseases

Cardiovascular disease is a leading cause of death in the world. Aging is associated with impaired vascular function due to endothelial dysfunction and altered redox balance, thus increasing the risk of cardiovascular diseases [37, 38]. Resveratrol could decrease atherosclerosis and improve cardiovascular health. For instance, an *in vitro* study found that senescent endothelial cells and aortas derived from old Wistar Kyoto rats demonstrated higher levels of superoxide production and oxidative stress as well as a lower level of bioactive nitric oxide (NO) than those from their young controls. Resveratrol could reduce superoxide generation, enhance NO level, and improve oxidative stress, thus protecting against aging-associated vascular diseases [39]. Another *in vitro* study carried on human umbilical vein endothelial cells showed that pretreatment with resveratrol could enhance the cell viability and superoxide dismutase (SOD) levels, reverse the elevated levels of senescence-associated *β*-galactosidase and intracellular ROS induced by H_2_O_2_ treatment, and upregulate autophagy, thus delaying the aging process of human umbilical vein endothelial cells [40]. Furthermore, methylglyoxal incubation significantly inhibited endothelium-dependent vasodilatation and decreased the expression of endothelial nitric oxide synthase (eNOS) in thoracic aorta of aged Wistar rats *in vitro*, while resveratrol treatment could improve methylglyoxal-induced endothelial dysfunction by increasing eNOS expression and activity [41]. In another study, cardiotoxicity was induced by doxorubicin in senescence-accelerated mice and led to a disturbance of ubiquitin-specific protease 7-related catabolic/proapoptotic signaling in hearts. The administration of resveratrol attenuated these adverse changes in aged hearts with the absence of Sirt1 inhibitors, like sirtinol, indicating that resveratrol exerted a cardioprotective effect through restoring the activity of Sirt1 [42]. In addition, resveratrol treatment significantly lowered aorta media thickness, inflammation, fibrosis, and oxidative stress in aged male C57BL/6 mice compared to the control group, protecting against arterial aging through modulating the activity of the renin-angiotensin system [43]. Moreover, a study reported that mitochondrial aldehyde dehydrogenase 2 (ALDH2) could exacerbate aging-induced cardiac remodeling and contractile dysfunction, which were mitigated by resveratrol treatment [44].

In conclusion, resveratrol exerted a cardioprotective effect mainly through enhancing the production of NO, modulating the activity of the renin-angiotensin system, ameliorating oxidative stress, and restoring the activity of Sirt1.

### 3.3. Effect of Resveratrol on Sarcopenia

Sarcopenia is an age-related syndrome characterized by the progressive loss of muscle mass and function [45, 46]. Many studies showed that resveratrol effectively improved the mass and function of skeletal muscle and decreased the happening of sarcopenia. In a study, aged male Fischer 344 x Brown Norway rats received different interventions (resveratrol, calorie restriction, or resveratrol combined with calorie restriction) to evaluate their efficacy of protecting against sarcopenia. The results indicated that short-term moderate resveratrol, calorie restriction, or resveratrol combined with calorie restriction could modestly alter key mitochondrial regulatory and apoptotic signaling pathways in the glycolytic muscle, which protected against aging-induced muscle loss [47]. Moreover, another study found that resveratrol treatment did not attenuate the decrease in the plantaris muscle wet weight during hindlimb suspension (creating disuse condition) in aged rats, but it could improve muscle mass during reloading after hindlimb suspension. Resveratrol enhanced the fiber cross-sectional area of type IIA and IIB fibers and promoted myogenic precursor cell proliferation in response to reloading after hindlimb suspension [48].

There was evidence that resveratrol in combination with exercise had a better effect on sarcopenia. A study found that aged mice treated with resveratrol combined with exercise training showed stronger muscle strength and endurance performance than with resveratrol or exercise training alone [49]. Another study showed that resveratrol supplementation or exercise training improved physical endurance in aged mice through increasing mitochondrial biogenesis and function, and their combination displayed better results [50]. Furthermore, resveratrol, exercise, or their combination significantly increased the relative grip strength and muscle mass in aged rats and reduced the increment in sarcomere length, I-band, and H-zone via antiapoptotic signaling pathways through activation of adenosine 5′-monophosphate-activated protein kinase (AMPK)/Sirt1 [51].

In brief, resveratrol could protect against sarcopenia, and its combination with exercise had a better effect. The effect might be mediated by the inhibition of apoptosis, the promotion of mitochondrial biogenesis and function, and the activation of Sirt1.

### 3.4. Effect of Resveratrol on Cancers

Age is reported to be one of the most important risk factors in the occurrence and development of cancers [52]. Considerable researches elucidated that resveratrol treatment could suppress the formation of cancer via inhibiting cell proliferation. An *in vitro* study showed that resveratrol significantly inhibited the proliferation, migration, and invasion of ovarian cancer cells, meanwhile damaging glycolysis, evoking apoptosis in these cells via increasing the expression and activation of AMPK and caspase 3, and decreasing the expression and activation of AMPK downstream kinase mammalian target of rapamycin (mTOR). In addition, an *in vivo* study manifested that resveratrol suppressed ovarian cancer growth and liver metastasis in the xenograft mouse model [53]. In another study, resveratrol could inhibit cell growth and proliferation in human gastric cancer SNU-601 cells by suppressing the activity of proviral integration site for Moloney murine leukemia virus-1 (PIM-1) kinase [54].

Several studies found that resveratrol could inhibit tumor progression by suppressing cell metastasis. In a study, prostate cancer cells cultured with resveratrol showed a decreased level of epithelial-mesenchymal transition- (EMT-) related proteins and inhibited cell migration and cell growth through the nuclear-factor kappa B (NF-*κ*B) pathway [55]. In another study, the effects of resveratrol on cell invasion and metastasis as well as changes in the expression of EMT markers and Akt/glycogen synthase kinase-3 *β* (GSK-3 *β*)/Snail signaling pathway were investigated in colon cancer cells. Furthermore, an *in vivo* lung metastasis model of colon cancer was developed in mice to investigate the effects of resveratrol on lung metastasis in colon cancer. *In vitro* and *in vivo* results indicated that resveratrol significantly inhibited cell migration and invasion in colon cancer through the reversal of EMT via the Akt/GSK-3 *β*/Snail signaling pathway [56].

The induction of apoptosis also contributed to the anticancer capacity of resveratrol. In a study, results showed that resveratrol significantly inhibited cell viability, induced apoptosis, and decreased cyclooxygenase-2 and prostaglandin receptor expression in human colon cancer cell lines [57]. Moreover, another *in vitro* study reported that resveratrol induced apoptosis in prostate cancer cells in a dose-dependent manner. At the molecular level, resveratrol suppressed the expression of androgen receptor protein as well as Akt phosphorylation [58].

To sum up, many studies showed that resveratrol was effective in the prevention and treatment of cancers mainly through inhibition of cell proliferation, induction of cell apoptosis, and suppression of cell migration.

### 3.5. Effects of Resveratrol on Other Diseases

Except for the above mentioned, some diseases were also closely associated with aging, like infertility and osteoporosis, and resveratrol was able to ameliorate these age-related diseases.

Aging leads to the loss of oocytes and follicles and impairs the quality of oocytes, which promotes age-associated ovarian aging and infertility [59]. In an *in vitro* study, the effects and mechanisms of resveratrol on postovulatory aging mouse oocytes were investigated. The results showed that resveratrol treatment significantly improved the quality of the oocytes and increased the ratios of fertilization and blastocyst via maintaining mitochondrion distribution and the normal morphology of spindle, alleviating oxidative stress, ameliorating apoptosis, and decreasing the loss of sperm binding [60]. In another study, after long-term (12 months) administration of resveratrol, female C57BL/6 mice reserved the ability of reproduction and showed a larger follicle pool than age-matched controls. Resveratrol significantly improved the number and quality of oocytes, telomerase activity, telomere length, and age-related gene expression in ovaries of mice [61].

Osteoporosis is a common disease of old age, characterized by the decrease of bone density and mass, the destruction of bone microstructure, and the increase of bone brittleness [62]. A study showed that resveratrol increased bone volume, bone trabecular number, and cortical thickness and reduced bone trabecular spacing in aged male Wistar rats [63]. Moreover, another study revealed that resveratrol accelerated osteoblast activity and bone growth and promoted the bone formation of C57Bl/6 mice in a Sirt1-dependant way [64].

Collectively, resveratrol showed abilities in the prevention and treatment of several age-related diseases, such as neurodegenerative diseases, cardiovascular diseases, sarcopenia, cancers, infertility, and osteoporosis.

## 4. Mechanisms of Resveratrol on Aging

**Figure 2:**
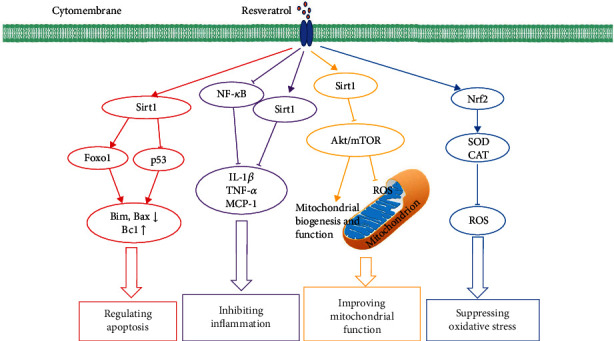
The mechanisms of resveratrol against aging. Resveratrol could stimulate the activity of nuclear factor erythroid-2 related factor 2 (Nrf2) and promote the activities of antioxidant enzymes, like superoxide dismutase (SOD) and catalase (CAT) to inhibit the production of reactive oxygen species (ROS), thus suppressing oxidative stress. Resveratrol could activate antiaging factor sirtuin1 (Sirt1) and downregulate the Akt/mTOR pathway to inhibit ROS in mitochondria and increase mitochondrial biogenesis and function, which could improve mitochondrial function. Resveratrol could promote the activities of nuclear-factor kappa B (NF-*κ*B) and Sirt1 to decrease the levels of inflammatory markers, like interleukin-1*β* (IL-1*β*), tumor necrosis factor-*α* (TNF-*α*), and monocyte chemoattractant protein-1 (MCP-1) to inhibit inflammation. Resveratrol could upregulate Sirt1, subsequently promote forkhead box protein O1 (Foxo1), and inhibit p53, thereby modulating the levels of apoptotic proteins Bim and Bax and antiapoptotic protein Bcl, which could regulate apoptosis.

Aging is the result of many factors on the body. The accumulation of oxidative stress, low-grade inflammation, and cell apoptosis is the prominent characteristic in the aging progress [72, 73]. Additionally, increasing evidence shows that mitochondrial dysfunction and gut microbiota imbalance contribute to the aging progress [74, 75]. Hence, we discuss the mechanisms of the antiaging effect of resveratrol as below (Table 3 and Figure 2).

### 4.1. The Suppression of Oxidative Stress

Oxidative stress is a crucial contributor to the aging process as well as the occurrence and development of age-related diseases. Lipid peroxidation, protein peroxidation, and an impaired defense system induced by excessive ROS gradually compromise cell structures and functions, eventually giving rise to cell senescence and accelerating the aging process [76, 77]. Many studies showed that resveratrol could inhibit the formation of oxidative stress, thus exerting an antiaging effect. For example, an *in vitro* study carried out on erythrocytes from healthy humans demonstrated that resveratrol treatment could activate the plasma membrane redox system and ascorbate free radical reductase in a dose-dependent manner, protect against lipid peroxidation and protein carbonylation, and recover the cellular redox homeostasis during aging [78]. An *in vitro* study also found that resveratrol treatment reduced the generation of ROS in immortalized lymphocytes from Alzheimer's disease patients or healthy controls, as well as upregulated the gene expression of antioxidant enzymes, like catalase (CAT), SOD, and antiaging factors, like Sirt1 and Sirt3 [79]. In addition, a study found that markers of oxidative damage to DNA, lipid oxidation, and protein oxidation age-dependently accumulated in the majority of mouse tissues, but chronic resveratrol treatment (6 or 12 months) could reverse the damage caused by oxidative stress. However, it also found that a 12-month resveratrol intake caused a significant increase in these oxidative damage markers in the kidney, suggesting that chronic resveratrol treatment might result in nephrotoxicity; hence, attention should be paid to the safe dosage and duration of resveratrol consumption [80].

The grey mouse lemurs were assigned to different groups to receive the standard-fed control diet, 30% fewer calories than the standard-fed control diet, and the standard-fed control diet supplemented with 200 mg/kg resveratrol for 3, 9, 15, and 21 months, to compare the effects of resveratrol and calorie restriction on oxidative stress. The results showed that oxidative stress age-dependently accumulated in grey mouse lemur, while both resveratrol and calorie restriction effectively ameliorated oxidative stress [81]. Another study evaluated the effects of resveratrol and exercise on endogenous antioxidant activities in livers of different-age mice. Aging induced the accumulation of oxidative damage in the liver, particularly impairing the glutathione-dependent system. Both resveratrol and exercise reversed the impact of aging on antioxidant capacity and sustained high activities of glutathione (GSH), glutathione peroxidase (GPx), and GSH transferase in old mice [82]. Moreover, oral administration of 10 mg/kg body weight resveratrol decreased the level of NO and retarded the lipid peroxidation in the cardiac tissue of male Wistar rats during the aging process, but the activities of CAT and SOD in the resveratrol intervention group had no significant difference from the controls, indicating that the antioxidant ability of resveratrol in the cardiac tissue might not result from the activation of CAT and SOD [83].

### 4.2. The Inhibition of Inflammation

A chronic low-grade inflammation level commonly exists in the aging process, which is known as inflammaging. Increasing evidence has highlighted the importance of inflammaging in the progress of aging and age-related metabolic diseases, and it is an extraordinary crucial risk factor for morbidity and mortality in older people [84, 85]. There are findings that suggest that resveratrol exerted an antiaging effect though improving inflammatory response. An *in vitro* study reported that treatment with resveratrol reversed most of the age-related changes in the secretory phenotype of vascular smooth muscle cells derived from aged Macaca mulatta. Resveratrol notably reduced the secretion of proinflammatory cytokines, such as interleukin- (IL-) 1*β*, IL-8, tumor necrosis factor-*α* (TNF-*α*), and monocyte chemoattractant protein-1 (MCP-1) in aged vascular smooth muscle cells. It also decreased the production of O_2_^·-^ in mitochondria and upregulated the transcriptional activity of nuclear factor erythroid-2 related factor 2 (Nrf2) [86]. Another *in vitro* study developed a model for cultured hippocampal astrocytes from newborn, adult, and aged Wistar rats and found that with increasing age, the levels of proinflammatory cytokines increased, while antioxidant defenses decreased in hippocampal astrocytes. Resveratrol treatment significantly lowered the level of proinflammatory cytokines TNF-*α* and IL-1*β* and improved antioxidant defenses [87].

In a study, neuroinflammatory response and cognitive deficits were induced by intraperitoneal injection of lipopolysaccharide in adult (3-6 months) and aged (22-24 months) mice. Aged mice showed being more prone to peripheral immune stimulation than adult mice. The dietary supplementation of resveratrol mitigated inflammatory response and cognitive deficits caused by lipopolysaccharides in aged mice and reduced the increase of IL-1*β* in plasma and the hippocampus [88]. In another study, peroxisome proliferator-activated receptor-*γ* coactivator-1*α* (PGC-1*α*) knockout and wild-type mice were randomly assigned to different groups intervened with or without resveratrol/exercise for 12 months, to analyze whether exercise and resveratrol inhibit age-associated low-grade inflammation in a PGC-1*α*-dependent manner. The results showed that long-term exercise training prevented an age-associated increase in the inflammatory response in a PGC-1*α*-dependent manner, while resveratrol supplementation reduced age-associated inflammation independently of PGC-1*α* [89]. Furthermore, metabolic stress was induced by a high-fat diet, leading to inflammation and cognitive disturbances in aged C56/BL6 mice (24 months). Resveratrol treatment reversed the increased levels of some proinflammatory cytokines, like TNF-*α*, IL-1, and IL-6, as well as other adverse changes in aged mice [90].

### 4.3. The Improvement of Mitochondrial Function

As a key factor in the metabolism of aerobic organisms, mitochondria are essential not only for obtaining ATP from glucose and fatty acids but also in many other essential functions in cells, including amino acid metabolism, pyridine synthesis, phospholipid modifications, and calcium regulation [91]. It is widely acknowledged that mitochondrial dysfunction and decreased mitochondrial content are hallmarks of aging and play an important role in promoting aging [92, 93]. Increasing literature reported that resveratrol regulated the mitochondrial function, thus delaying the aging process. An *in vitro* study showed that resveratrol treatment increased the expression of Sirt1 and evoked autophagy in both oocytes and granulosa cells derived from aged cows. Resveratrol also increased the mitochondrial DNA (mtDNA) copy numbers and the ATP content in oocytes and promoted oocytes to develop into the blastocyst stage, thus improving the quality of oocytes. All of these effects were associated with the regulation of mitochondrial biogenesis and degradation [94]. In addition, an *in vivo* study showed that aged mice receiving resveratrol and/or exercise-training for 4 weeks showed a markedly longer time to exhaustion with lower blood lactate and free fatty acids levels and an improved oxidative status with decreased gastrocnemius muscle lipid peroxidation and increased activities of antioxidant enzymes (like CAT and SOD). The improvement of physical endurance and oxidative stress was associated with the regulation of mitochondrial biogenesis and function [50]. Furthermore, an *in vivo* study demonstrated that short-term administration of resveratrol significantly improved mitochondria function and alleviated oxidative stress-induced damage in postovulatory aging oocytes of middle-aged mice. Resveratrol treatment also slowed down the aging-induced oocyte deterioration, upregulated the expression of the antiaging molecule Sirt1, decreased the level of ROS, and prevented apoptosis, showing a multifactor effect on aging [95]. Another *in vivo* study showed that mtDNA integrity, mtDNA copy number, mitochondrial fusion regulators, mitophagy, and the expression of antioxidant-related genes were all decreased in zebrafish retinas upon aging, whereas Akt/mTOR activity and inflammation were increased. Resveratrol treatment could improve mitochondrial quality and function, as well as the downregulated Akt/mTOR pathway in zebrafish retinas to reverse the age-dependent changes [17].

### 4.4. The Regulation of Apoptosis

As a consequence of intracellular or extracellular damage, apoptosis is activated as an adaptive response for the maintenance of homeostasis. On the one hand, apoptosis contributed to the disappearance of nonfunctional and damaged cells, such as cancer cells. On the other hand, its dysregulation played an essential role in the development of age-related pathologies. For example, excessive neuronal apoptosis promoted the development of neurodegenerative diseases [96, 97]. Many studies showed that resveratrol could regulate apoptosis to protect against aging and age-related diseases. In an *in vivo* study, impairment of learning and memory ability in aged rats was induced by sevoflurane and nitrous oxide, accompanied with neuronal apoptosis, but pretreatment with resveratrol modified the performance of learning and memory and suppressed neuronal apoptosis by upregulating the expression of Sirt1 in aged rats [67]. Moreover, aged senescence-accelerated mice showed the decreased activities of antiapoptosis and antioxidants as well as mRNA expressions of Sirt1 with the aging process and the increased levels of inflammatory response and NF-*κ*B protein expression. Resveratrol administration could improve apoptotic, proinflammatory, and prooxidant statuses and increase Sirt1 mRNA expression, as well as decrease NF-*κ*B expression, thus exerting an antiaging effect [16]. Additionally, a study compared the antiaging effects of resveratrol and caloric restriction. The results showed that both resveratrol and caloric restriction displayed antiaging activities by inhibiting senescence and apoptosis and recovering cognitive impairment and oxidative damage. The 10 *μ*M resveratrol *in vitro* and the high-dose group (100 mg/kg body weight) *in vivo* showed more powerful effects on antiaging and stimulating the Sirt1 level than caloric restriction [98]. In a study, Sprague-Dawley rats were randomly distributed to the sham control group, aging model group, and aging rats with different treatment groups (resveratrol, soy isoflavones, resveratrol combined with soy isoflavones, and estrogen replacement therapy). The results showed that the increased apoptotic index, elevated mitochondrial swelling and vacuolation, accelerated oxidative stress, and decreased mitochondrial integrity were observed in the aging model group. All of the treatment groups could markedly decrease the apoptotic index, improve mitochondrial function, and reduce oxidative stress. The combination of resveratrol and soy isoflavones exerted a stronger effect than that in the alone administration [99]. In another study, aging significantly impaired exercise capacity and voluntary motor behavior and elevated the contents of p53, proapoptotic protein, and apoptotic DNA fragmentation in aged rats, while long-term resveratrol treatment could improve physiological performance and increase the expression of the antiapoptotic protein in aged rats via the activation of Sirt1 deacetylase activity [100].

### 4.5. The Modulation of Gut Microbiota

In recent years, the effects of gut microbiota on health have attracted increasing attention [101]. Accumulating studies have shown that natural products could play an important role in modulating the homeostasis of gut microbiota, thus protecting against many diseases [102–105]. As shown in some studies, resveratrol treatment markedly regulated the composition and metabolic function of gut microbiota in high-fat diet-induced obese mice, exerting an antiobesity ability [106–108]. Some studies also showed that resveratrol could protect against diabetic nephropathy, hepatic steatosis, and hypertension through the regulation of gut microbiota [109–111]. However, there is little evidence about the effect of resveratrol on antiaging via targeting with gut microbiota; hence, it is of great significance to investigate whether resveratrol could stave off the process of aging via an intestinal flora regulatory way, which represents a novel target for interventions.

In conclusion, resveratrol exerted an antiaging action through the suppression of oxidative stress, inhibition of inflammatory response, modulation of mitochondrial function, and regulation of apoptosis. Considering the importance of gut microbiota in the maintenance of health, the antiaging effect of resveratrol targeting the regulation of gut microbiota should be studied in the future.

## 5. Clinical Trials

Several clinical trials studied the antiaging effect of resveratrol. For example, 200 mg/d resveratrol intake enhanced memory performance accompanied with improved glucose metabolism and hippocampal functional connectivity in 23 healthy overweight older adults, exerting beneficial effects on brain aging [118]. In addition, the effect of a cream containing trans-resveratrol on human volunteers was evaluated by measuring different skin parameters, and all patients showed a visible improvement of clinical conditions with a significant decrease of aging signs [119]. However, a clinical trial showed that resveratrol in combination with exercise could abolish the positive effects of exercise on cardiovascular health in aged men, and the negative effects of resveratrol supplementation on training-induced benefits were partly related to the antioxidant properties of this compound [120]. In the future, more clinical trials are necessary to confirm the effectiveness of resveratrol and observe its possible risks, such as nephrotoxicity [80].

## 6. Conclusions

Many studies show that resveratrol has tremendous potential in the prevention and treatment of aging. Accumulating studies revealed that resveratrol could markedly extend lifespan and protect against a series of age-related diseases, such as neurodegenerative diseases, cardiovascular diseases, sarcopenia, cancers, infertility, and osteoporosis. Some clinical trials also showed the antiaging effect of resveratrol. Several mechanisms contributed to the antiaging effect of resveratrol, mainly including the suppression of oxidative stress, the inhibition of inflammation, the regulation of mitochondrial function, and the regulation of apoptosis. In the future, studies about whether resveratrol could protect against aging through the modulation of gut microbiota should be carried out. In addition, the synergetic effects of resveratrol with other natural compounds should be studied to guide future product development and reduce the cost of new formulations. Moreover, effects of some fruits and vegetables on lifespan should be evaluated, such as grape, peanut, blueberry, cucumber, tomato, red cabbage, and spinach, because they have relatively high contents of resveratrol. Furthermore, more clinical trials are necessary to confirm the effectiveness of resveratrol, and attention should also be paid to the safe dosage and duration of resveratrol consumption since chronic resveratrol treatment might result in nephrotoxicity.

## Figures and Tables

**Table 1:** Effect of resveratrol on life extension.

Study type	Subjects	Administration methods	Dose & duration	Main effects	Ref.
*In vitro*, *in vivo*	Human cells, *Caenorhabditis elegans*	Culture	100 *μ*M	Induced autophagy in human cells and *Caenorhabditis elegans*, prolonged lifespan in *Caenorhabditis elegans* through the Sirt1-dependent induction of autophagy	[23]
*In vivo*	Honey bees	Administered in candy and syrup	10 ppm	Inhibited *Nosema ceranae* infection and prolonged lifespan of honey bees	[24]
*In vivo*	*Drosophila melanogaster*	Diet supplement	25-800 *μ*M	Extended adult longevity in both the male and female flies by scavenging ROS and neuroprotection	[25]
*In vivo*	Rats	Diet supplement	NA	Delayed vascular aging, but did not extend lifespan	[29]
*In vivo*	*Nothobranchius guentheri* (fish)	Diet supplement	200 *μ*g/g food	Prolonged longevity, improved cognitive ability and aging-related histological markers	[27]
*In vivo*	*Drosophila melanogaster*	Culture	NA	Prolonged longevity, ameliorated age-related symptoms	[26]
*In vivo*	HtrA2 knockout mice	Oral administration	25 mg/kg BW	Prolonged longevity, delayed worsening of the motor phenotype	[28]

Note: NA: not available; ROS: reactive oxygen species; BW: body weight.

**Table 2:** Effect of resveratrol on age-related diseases.

Study type	Subjects	Administration methods	Dose & duration	Effects and mechanisms	Ref.
*Neurodegenerative diseases*					
*In vivo*	C57BL/6J mice	Intraventricular injection	5 *μ*g/*μ*L for 1 week	Improved learning and memory functions in a Sirt1-dependent way	[33]
*In vivo*	Aged C57BL/6 mice	Oral administration	200 mg/kg for 10 days	Rescued cortical neurovascular coupling responses to improve neuronal activity and function by restoring cerebromicrovascular endothelial function and decreasing ROS production	[65]
*In vivo*	C57BL/6 mice	Intraperitoneal injection	100 mg/kg for 7 days	Alleviated the hippocampus-dependent cognitive impairment via anti-inflammation and antiapoptosis actions	[66]
*In vivo*	Male F344 rats	Intraperitoneal administration	40 mg/kg for 4 weeks	Improved memory and mood functions, increased hippocampal neurogenesis and microvasculature, and reduced glial activation	[34]
*In vivo*	Old male rats	Chronic administration	20 mg/kg for 4 weeks	Improved cognitive impairment by enhancing the secretion of neurotransmitters (serotonin, noradrenaline, and dopamine), which is largely due to the increased activities of TPH and TH	[35]
*In vitro*, *in vivo*	Adult hippocampal precursor cells; female Balb/C mice	Cell culture; injection	40 mg/kg for 2 weeks	Induced neuronal differentiation in adult hippocampal precursor cells without effects on proliferation *in vitro*, improved behavioral performance, increased production of new neurons, elevated population of doublecortin-expressing intermediate cells, and promoted hippocampal neurogenesis *in vivo* through the phospho-Akt and phosphoprotein kinase C signaling pathways	[32]
*In vivo*	Aged Sprague-Dawley rats	Intraperitoneal injection	100 mg/kg for 7 days	Inhibited neuronal apoptosis and improved behavioral performance via Sirt1-p53 signaling pathway	[67]
*In vivo*	Male Wistar rats	Oral administration	50 mg/kg for 12 weeks	Improve aging-induced emotional and spatial learning memory impairment via inhibiting inflammation	[36]
*In vitro*, *in vivo*	SH-SY5Y neuroblastoma cells; male C57BL/6 mice	Cell culture; feeding	1 or 5 *μ*M; 120 mg/kg	Alleviated age-related motor decline and exerted neuroprotection via the promotion of dopamine neuronal survival and activation of the ERK1/2 pathways	[68]
*Cardiovascular disease*					
*In vitro*	Aging endothelial cells; aortas of old WKY rats	Cell and tissue culture	10 *μ*mol/L	Inhibited S6K1 signaling, reduced superoxide generation, and enhanced NO levels	[39]
*In vivo*	Senescence-accelerated mice prone 8 (SAMP8)	Intraperitoneal injection	20 mg/kg for 3 days	Attenuated doxorubicin-induced cardiotoxicity through restoring the activity of Sirt1	[42]
*In vivo*, *in vitro*	Aged male C57BL/6 mice; vascular smooth muscle cells	Dietary supplementation	40 mg/kg for 6 months	Lowered aorta media thickness, inflammation, fibrosis, and oxidative stress and protected against arterial aging through modulating the activity of the renin-angiotensin system	[43]
*In vivo*	Wistar albino rats	Drinking water	0.05 mg/mL for 6 weeks	Altered vessel responsiveness and biomarkers related to vascular functions	[69]
*In vitro*	Human umbilical vein endothelial cells	Cell culture	10 *μ*M	Enhanced the cell viability and SOD levels, inhibited the increased levels of senescence-associated *β*-galactosidase and intracellular ROS induced by H_2_O_2_ treatment, and upregulated autophagy	[40]
*In vitro*	Thoracic aorta of aged Wistar rats	Organ culture	30 *μ*M for 4 h or 24 h	Improved methylglyoxal-induced endothelial dysfunction by increasing eNOS expression and activity	[41]
*Sarcopenia*					
*In vivo*	Fischer 344 x Brown Norway rats	Dietary supplementation	50 mg/kg for 6 weeks	Protected against aging-induced muscle loss via modestly altered key mitochondrial regulatory and apoptotic signaling pathways in glycolytic muscle	[47]
*In vivo*	Fischer 344 x Brown Norway rats	Oral gavage	125 mg/kg	Improved muscle mass, increased the fiber cross-sectional area of type IIA and IIB fibers during reloading after hindlimb suspension due to decreases in the abundance of proapoptotic proteins	[48]
*In vivo*	Aged C57BL/6J mice	Oral gavage	25 mg/kg BW for 4 weeks	Resveratrol combined with exercise training showed stronger muscle strength and endurance performance of aged mice than the resveratrol or exercise training alone	[49]
*In vivo*	Aged male Sprague-Dawley rats	Dietary supplementation	150 mg/kg for 6 weeks	Increased the relative grip strength and muscle mass and reduced the increment in sarcomere length, I-band, and H-zone via antiapoptotic signaling pathways through the activation of AMPK/Sirt1	[51]
*In vivo*	C57/BL6 mice	Dietary supplementation	0.04% for 6 months	Inhibited tubular aggregates and showed better resistance to fatigue	[70]
*In vivo*	C57BL/6J male mice	Dietary supplementation	0.04% for 6 months	Showed a better fatigue resistance	[71]
*Cancers*					
*In vitro*	Ovarian cancer cells	Cell culture	25-800 *μ*M	Suppressed proliferation and evoked apoptosis via inhibiting glycolysis and targeting AMPK/mTOR signaling pathway	[53]
*In vitro*	Gastric cancer cell	Cell culture	0, 25, 50, and 100 *μ*M for 96 h	Inhibited cell proliferation and survival through inhibition of PIM-1 kinase activity	[54]
*In vitro*	Human prostate cancer cell lines	Cell culture	50 *μ*M for 48 h	Inhibited cell proliferation and migration through the NF-*κ*B pathway	[55]
*In vitro*, *in vivo*	Colon cancer cells; nude mice	Cell culture; intraperitoneal injection	0-240 *μ*mol/L; 150 mg/kg BW	Inhibited invasion and metastasis through the reversal of EMT via the Akt/GSK-3 *β*/Snail signaling pathway	[56]
*In vitro*	Human colon cancer cell lines	Cell culture	0-50 *μ*M	Inhibited cell viability, induced apoptosis, and decreased expression of cyclooxygenase-2 and prostaglandin receptor	[57]
*In vitro*	Prostate cancer cells	Cell culture	25-100 *μ*M	Inhibited proliferation and promoted apoptosis	[58]
*Other diseases*					
*In vitro*	Mouse oocytes	Cell culture	1 *μ*M	Improved the quality of postovulatory aging oocytes via maintaining mitochondrion distribution and the normal morphology of spindle, alleviating oxidative stress, ameliorating apoptosis, and decreasing the loss of sperm binding	[60]
*In vivo*	Female C57BL/6 mice	Drinking water	30 mg/L for 6 or 12 months	Reserved the ability of reproduction and showed a larger follicle pool, improved the number and quality of oocytes, telomerase activity, telomere length, and age-related gene expression in ovaries	[61]
*In vivo*	Aged male Wistar rats	Drinking water	10 mg/kg for 10 weeks	Increased bone volume, bone trabecular number, and cortical thickness and reduced spacing between trabeculae	[63]
*In vivo*	C57BL/6 mice	Dietary supplementation	300 mg/kg for 10 weeks	Accelerated osteoblast activity and bone growth, and promoted bone formation in a Sirt1-dependant way	[64]

Note: Sirt1: sirtuin1; ROS: reactive oxygen species; TPH: tryptophan hydroxylase; TH: tyrosine hydroxylase; Akt: protein kinase B; ERK1/2: extracellular-regulated kinases 1 and 2; S6K1: ribosomal protein S6 kinase, polypeptide1; NO: nitric oxide; SOD: superoxide dismutase; eNOS: endothelial nitric oxide synthase; AMPK: 5′-monophosphate-activated protein kinase; mTOR: mammalian target of rapamycin; PIM-1 kinase: proviral integration site for Moloney murine leukemia virus-1 kinase; BW: body weight; NF-*κ*B: nuclear-factor kappa B; EMT: epithelial-mesenchymal transition; Akt/GSK-3 *β*/Snail: protein kinase B/glycogen synthase kinase-3 *β*/Snail signaling.

**Table 3:** Effects and mechanisms of resveratrol on aging.

Study type	Subjects	Administration methods	Dose & duration	Effects and mechanisms	Ref.
*The suppression of oxidative stress*					
*In vitro*	Human erythrocytes	Cell culture	0.1–100 *μ*M	Activated the plasma membrane redox system and ascorbate-free radical reductase, protected against lipid peroxidation and protein carbonylation, and restored the cellular redox homeostasis	[78]
*In vitro*	Immortalized lymphocytes	Cell culture	10 and 50 *μ*M	Reduced the generation of ROS, upregulated the gene expression of antioxidants and antiaging factors	[79]
*In vivo*	F2 four-way cross-hybrid mice	Drinking water	14.09 mg/L for 6 or 12 months	Reversed oxidative damage but might result in nephrotoxicity	[80]
*In vivo*	Male grey mouse lemur	Diet supplement	200 mg/kg for 3-21 months	Ameliorated oxidative stress with age increase	[81]
*In vivo*	Male C57BL/6J mice	Drinking water	500 *μ*g/animal for 6 months	Retarded the impact of aging and sustained high activities of GSH, GPx, and GSH transferase activities	[82]
*In vivo*	Male Wistar rats	Oral administration	10 mg/kg for 2-8 months	Decreased the level of NO and retarded the lipoperoxidation in the cardiac tissue	[83]
*In vivo*	C57BL/6 mice	Diet supplement	0.05% for 10 days	Blunted the exercise-induced increase in xanthine oxidase activity in muscles, lower H_2_O_2_, and Nox4 protein levels, increased the ratio of reduced GSH to oxidized GSH, prevented the increase in lipid oxidation, increased CAT and SOD activities	[112]
*In vivo*	Young and aged rats	Perfusion	NA	Ameliorated H_2_O_2_-induced oxidative stimulus in both young and aged rat brains and ameliorated basal oxidative stress in aged rat brains	[113]
*In vivo*	Aged C57BL/6 mice	Oral administration	30 mg/kg	Ameliorating renal oxidative stress via the Sirt1-mediated klotho expression	[114]
*The inhibition of inflammation*					
*In vitro*	Vascular smooth muscle cells	Cell culture	1 *μ*M	Reduced the secretion of IL-1*β*, IL-8, TNF-*α*, and MCP-1, decreased the production of O_2_^·-^ in mitochondria, and upregulated the transcriptional activity of Nrf2	[86]
*In vitro*	Hippocampal astrocyte	Cell culture	10 *μ*M	Decreased proinflammatory cytokines IL-1*β* and TNF-*α* and increased antioxidant defenses	[87]
*In vivo*	Male BALB/c mice	Diet supplement	0.4% for 4 weeks	Mitigated inflammatory response and cognitive deficits and reduced the increase of IL-1*β* in plasma and the IL-1*β* mRNA in the hippocampus	[88]
*In vivo*	Female C57BL/6 mice	Diet supplement	4 g/kg for 12 months	Reduced age-associated inflammation independently of PGC-1*α*	[89]
*In vivo*	Aged female mice	Oral gavage	0.1 mg/kg for 10 days	Attenuated peripheral and brain inflammation and ischemic brain injury	[115]
*In vivo*	Male C57BL/6J mice	Diet supplement	1 g/kg, *W*/*W*	Reduced the inflammation and cognitive disturbances induced by metabolic stress	[90]
*The improvement of mitochondrial function*					
*In vitro*	Oocytes and granulosa cells	Cell culture	20 *μ*M	Affected both oocytes and granulosa and improved the quality of oocytes through upregulation of mitochondrial biogenesis and degradation	[94]
*In vivo*	Aged mice	Oral gavage	15 mg/kg for 4 weeks	Improved physical endurance and oxidative stress via the regulation of mitochondrial biogenesis and function	[50]
*In vivo*	Female ICR mice	Intraperitoneal injection	50 mg/kg BW	Improved mitochondrial function, alleviated oxidative stress, and prevented apoptosis	[95]
*In vivo*	Aged zebrafish	Administration	20 mg/L	Promoted mitochondrial function and downregulated Akt/mTOR pathway activity	[17]
*The regulation of apoptosis*					
*In vivo*	Aged Sprague-Dawley rats	Intraperitoneal injection	100 mg/kg for 7 days	Modified the performance of learning and memory, suppressed neuronal apoptosis	[67]
*In vivo*	Aged senescence-accelerated mice	Drinking water	5 mg/kg	Modulated the inflammatory, oxidative, and apoptotic status related to aging	[16]
*In vivo, in vitro*	Male albino Wistar rats; human diploid fibroblast strain	Oral administration	50, 100 mg/kg	Displayed antiaging activities by inhibiting senescence and apoptosis and recovering cognitive impairment and oxidative damage	[98]
*In vitro*	Mouse neuronal N2a cells	Cell culture	1.5 to 25 *μ*M	Counteracted apoptosis, autophagy, and oxidative stress, associated with mitochondrial and peroxisomal dysfunction induced by 7-Ketocholesterol	[116]
*In vivo*	Sprague-Dawley rats	Oral gavage	80 mg/kg	Decreased apoptotic index, improved mitochondrial function, and inhibited oxidative stress	[99]
*In vivo*	Senescence-accelerated mice	Diet supplement	4.9 mg/kg for 8 months	Improved exercise capacity and voluntary motor behavior, increased the protein expression of antiapoptotic Bcl2	[100]
*In vivo*	Male senescence-accelerated mice	Intraperitoneal injection	20 mg/kg/day for 3 days	Attenuated the doxorubicin-induced elevations of apoptotic and catabolic markers measured as Bax, caspase 3 activity, apoptotic DNA fragmentation, ubiquitinated proteins, and proteasomal activity in aged muscles	[117]

Note: ROS: reactive oxygen species; GSH: glutathione; GPx: glutathione peroxidase; NO: nitric oxide; Nox4: NADPH oxidase 4; CAT: catalase; SOD: superoxide dismutase; Sirt1: sirtuin1; IL: interleukin; TNF-*α*: tumor necrosis factor-*α*; MCP-1: monocyte chemoattractant protein-1; Nrf2: nuclear factor erythroid-2 related factor 2; PGC-1*α*: peroxisome proliferator-activated receptor-*γ* coactivator-1*α*; Bcl2: B-cell lymphoma-2; Bax: BCL2-associated X.
